# Hematopoietic stem cell transplantation induces immunologic tolerance in renal transplant patients via modulation of inflammatory and repair processes

**DOI:** 10.1186/1479-5876-10-182

**Published:** 2012-08-31

**Authors:** Duojiao Wu, Guisheng Qi, Xuanchuan Wang, Ming Xu, Ruiming Rong, Xiangdong Wang, Tongyu Zhu

**Affiliations:** 1 Qingpu Branch, Fudan University Zhongshan Hospital, Shanghai, China; 2 Department of Urology, Fudan University Zhongshan Hospital, Shanghai, China; 3 Shanghai Key Laboratory of Organ Transplantation, Shanghai, China

**Keywords:** Kidney transplantation, Immunologic rejection, Immunologic tolerance, Hematopoietic stem cell transplantation, Proteome

## Abstract

**Background:**

Inducing donor-specific tolerance in renal transplant patients could potentially prevent allograft rejection and calcineurin inhibitor nephrotoxicity. Combined kidney and hematopoietic stem cell transplant from an HLA-matched donor is an exploratory and promising therapy to induce immune tolerance. Investigtion of molecular mechanisms involved in the disease is needed to understand the potential process of cell therapy and develop strategies to prevent this immunologic rejection.

**Methods:**

We enrolled nine patients in a clinical study in which cryopreserved donor hematopoietic stem cells were infused on days 2, 4, and 6 after kidney transplantation. One month post-transplant, 4 plasma samples were collected from combined transplants (C + Tx), and 8 plasma samples from patients with kidney transplantation alone (Tx). High abundance proteins in plasma were depleted and the two-dimensional liquid chromatography-tandem mass spectrometry coupled with iTRAQ labeling was utilized to identify the protein profiling between the two groups. Clusters of up- and down-regulated protein profiles were submitted to MetaCore for the construction of transcriptional factors and regulation networks.

**Results and Discussion:**

Among the 179 identified proteins, 65 proteins were found in C + Tx with at least a 2-fold change as compared with Tx. A subset of proteins related to the complement and coagulation cascade, including complement C3a,complement C5a, precrusors to fibrinogen alpha and beta chains,was significantly downregulated in C + Tx. Meanwhile, Apolipoprotein-A1(ApoA1), ApoC1, ApoA2, ApoE, and ApoB were significantly lower in Tx compared to C + Tx. Gene ontology analysis showed that the dominant processes of differentially expressed proteins were associated with the inflammatory response and positive regulation of plasma lipoprotein particle remodeling.

**Conclusions:**

Thus, our study provides new insight into the molecular events in the hematopoietic stem cell-induced immunologic tolerance.

## Introduction

Although renal transplantation is an effective therapy of end-stage renal failure, a significant proportion of organs are lost every year due to chronic allograft rejection and immunosuppressive drugs toxicity. The induction of immune tolerance has the potential to prevent this chronic rejection and drug toxicity. Immune tolerance of organ transplants has been induced in laboratory animals by infusing organ recipients with hematopoietic stem cells (HSC) from the organ donor before or after transplantation [1,2]. The continued presence of the organ donor’s immune cells in the recipient’s thymus and peripheral lymphoid tissue eliminates T-cell clones that could react to alloantigens of the graft, thereby promoting and maintaining immune tolerance [3,4].

At present, few clinical centers have addressed this issue. Thus, the molecular mechanisms of stem cell-induced immunologic tolerance in human remains unknown. A more thorough understanding of HSC transplantation-induced immune tolerance would lead to a more successful treatment of renal transplant patients. Our clinical study showed that the recipients of combined kidney and HSC transplants maintained stable kidney function with a decreased dose of immunosuppressive agents during the up to 29 months follow up. The present study uses iTRAQ labeling and quantitative proteomics technology to explore the potential mechanism involved in this cell therapy. We hypothesized that these new insights into the molecular basis of HSC-induced immunologic tolerance could be used to identify novel targets for the prevention of immunologic rejection.

## Materials and Methods

### Transplanted patients

The study had been registered in Chinese Clinical Trial Registry *(*http://www.chictr.org/*, ChiCTR-TNC-09000399)* and had obtained consents from patients of combined HSC and kidney transplantation. Potassium-EDTA plasma samples were collected by venipuncture from the 12 patients undergoing renal transplant. These patients included 8 kidney transplantation recipients with only organ transplantation (Tx) and 4 patients with combined HSC and kidney transplantation (C + Tx) at Zhongshan Hospital (Shanghai, China). Plasma samples were collected from each patient before surgery (Pre-Tx) and one month after surgery. The collected plasma was aliquoted and stored frozen at −80°C. All patient samples were obtained with informed consent and ethical approval by the Ethics Board of ZhongShan Hospital.

### Hematopoietic Cell Transplantation

Before collecting hematopoietic cells, the donor received a 5-day course of subcutaneous injections of granulocyte colony-stimulating factor (G-CSF) at a dose of 7.5 mg per kilogram per day. G-CSF mobilized donor mononuclear cells into the peripheral blood, and these cells were harvested by the com.tec blood cell separator (Fresenius AG, Germany). CD34^+^ and CD3^+^ cells were assessed by flow-cytometric analysis. One day before the transplantation, donor HSC were harvested and stored on −70°C. Total lymphoid irradiation was performed on day −3,-2 and −1. We used SIEMENS Linac (ONCOR), 6MV photon beam with multileaf collimator (MLC), a pattern of 480~510 cGy/3Fx/3D, 160~170 cGy/1Fx per single fraction. Patients received irradiation to all major lymphatic regions using anterior-posterior-posterior-anterior (AP/PA) fields. The supradiaphragmatic or “mantle” field encompassed the low cervical, supraclavicular, infraclavicular, axillary, mediastinal, and pulmonary hilar nodes, as well as the thymus [5,6]. During the operation, patients received 50 mg of rabbit antithymocyte globulin [7].

On post-operative day 2, 4, and 6, patients received an intravenous infusion of cryopreserved donor cells, which included CD34^+^ cells (0.20-3.0 × 10^6^ per kilogram). Patients were treated with a classic triple immunosuppressive regimen of tacrolimus/prednisone/mycophenolate mofetil or ciclosporin A/prednisone/mycophenolate mofetil. Microchimerism was determined by means of DNA genotyping of simple sequence-length polymorphic markers that encode short tandem repeats (AmpFl STR Identifiler PCR Amplification Kit, Applied Biosystems).

### Immunodepletion of high-abundance proteins

High abundance proteins in plasma were depleted using Agilent Multiple Affinity Removal Column - Human 14 (MARS) kit (Agilent Technologies) as described in our previous report [8].

### Protein digestion and labeling with iTRAQ reagents

The proteins of each sample were denatured, alkylated, and digested with sequencing-grade modified trypsin with a protein-to enzyme ratio of 20:1 at 37°C overnight and then labeled with the iTRAQ tags (Applied Biosystems, Warrington, UK) as follows: pre-transplantation patients −113 tag; C + Tx −115 tag; Tx −119 tag. The labeled digests were then mixed and dried. The iTRAQ technique allows the differential labeling of peptides from distinct proteomes [9,10].

### Off-line 2D LC-MS/MS

The combined peptide mixture was fractionated by strong cation exchange (SCX) chromatography on a 20 AD high-performance liquid chromatography (HPLC) system (Shimadzu, Kyoto, Japan). The concentrated iTRAQ labelled sample was added to loading buffer (10 mM KH2PO4 in 25% acetonitrile, pH 2.6) and loaded onto the column. Buffer A was identical in composition to the loading buffer, and buffer B was comprised of buffer A with 350 mM KCl. Separation was performed using a linear binary gradient of 0–80% buffer B in buffer A at a flow rate of 200 ml/min for 60 min. The absorbance at 214 nm and 280 nm was monitored and a total of 32 SCX fractions was collected along the gradient. These fractions were dried down by the rotary vacuum concentrator, dissolved in buffer C (5% acetonitrile, 0.1% FA) and analyzed on a QSTAR XL system (Applied Biosystems) interfaced with a 20 AD HPLC system (Shimadzu). Peptides were separated on a Zorbax 300SB-C18 column (Agilent Technologies). The HPLC gradient was 5–35% buffer B (95% acetonitrile, 0.1% FA) in buffer A (5%ACN, 0.1% FA) at a flow rate of 0.3 ml/min for 90 min. Survey scans were acquired from m/z 400–1800 with up to four precursors selected for MS/MS from m/z 100–2000. Each SCX fraction was analyzed in duplicate.

### Data analysis

The MS/MS spectra were extracted and searched against the Swissprot database (HUMAN 090210) using ProteinPilot software (version 3.0, revision 114732, Applied Biosystems). The following parameters were set in the searching: trypsin as enzyme, iTRAQ as sample type, no special factors, biological modification, thorough identification search, etc. Using the following criteria to consider a protein for further statistical analysis: unused ProtScore >1.3 with at least one peptide with 95% confidence per repetition. The candidate proteins were examined in the Protein ID of the Protein Pilot software. Protein expression ratios were computed on basis of the peak area ratios of the peptides accounting for the same protein. All of the normalized peptides of a protein were then averaged and the standard deviation of the mean was determined for each protein. The bias correction algorithm was applied to correct for unequal mixing during the combination of the different labeled samples, based on the assumption that most proteins do not change in expression. All quant ratios (both the average ratio for proteins and the individual peptide ratios) were corrected for the bias.

### Analysis of regulatory networks

MetaCore (GeneGo, St. Joseph, MI) is used for functional analysis of experimental data from high-throughput expression assays. The software has a curated database of human protein–protein and protein–DNA interactions, transcriptional factors, signaling, metabolism, and bioactive molecules. The software analyzes the input gene sets and develops a list of relevant biological networks that not only link the gene members in the clusters, but also provide the functionality to identify the co-regulated genes ranked by statistics and scores. All networks analyzed in this study were constructed by use of the default settings and values under analysis network option. To functionally annotate the differentially expressed proteins identified in this study, the proteins (≥2.0-fold change) were entered into GeneGo’s MetaCore for analysis. The genes encoding expressed overabundant proteins were used as the input list for generation of regulatory networks using Transcription Regulation algorithm which generated sub-networks centered on transcription factors. Sub-networks are ranked by a *P*-value and interpreted in terms of Gene Ontology. For every transcription factor (TF) with direct target(s) in the root list, this algorithm generated a sub-network consisting of all shortest paths to this TF from the closest receptor with direct ligand(s) in the root list.

### Technical validation

Five proteins were analyzed by ELISA based on commercially available kits following the manufacturers’ directions: Human ApoA1, ApoB, Fibrinogen (Abcam,UK), C3a, C5a (Hycult biotech, The Netherlands). All samples were analyzed in triplicates. The independent samples *t*-test was used to compare mean difference between patients of Tx and C + Tx groups. Mann–Whitney U tests were utilized to compare the medians of plasma concentration of C3a and C5a in patients with and without HSC. *P* value <0.05 is considered as significant. All of the statistical analysis was implemented using SPSS15.0 software.

## Results

The allograft function, the clinical course, and the allograft biopsy results of two groups were extracted as Table 1. In Tx group, patients received 7–34 months follow up. For most recipients, serum creatinine (SCr) was stable, ranging from 105 to 152umol/l at their most recent out-patient appointment. Three of the eight patients without cell therapy experienced acute rejection. Although only four patients with HSC transplantation were analyzed by proteomics study in the present paper, a total of nine cases were performed in our stem cell research (Additional file 1: Table S1). The patients with cell transplantation had no immunologic rejection during the six months following surgery. These patients received 1–29 months follow up. Although immunosuppressant dosages were gradually reduced, most patients maintained stable kidney function. There were no incidents of severe infection requiring hospitalization among these patients. Microchimerism was detected to evaluate the HSC transplant efficiency. For example, in patient 1, the microchimerism appeared in bone marrow 21 days after transplantation and peripheral blood 28 days after transplantation with a chimeric ranging from 30% to 50%. The microchimerism could also be detected in peripheral blood 42 days after transplantation, but vanished 6 months after transplantation. However, the rate of microchimerism in other patients without HSC transplant ranged from 1% to 5%.

**Table 1:** Clinical information of patients

**Variable**	**Kidney transplantation (n = 8)**	**Combined hematopoietic stem cells and kidney transplantation (n = 4)**
Recipient gender, n (% female)	37.50 %	0
Recipient age, mean ± SD	38.00 ± 12.70	33.00 ± 2.16
Creatinine at Tx [μmol/L], mean ± SD	2105.75 ± 318.69	1569.25 ± 458.77
Creatinine at one month after Tx [μmol/L], mean ± SD	127.00 ± 47.65	110.50 ± 10.89
Hemoglobin at Tx [g/L]	102 ± 32.32	104.00 ± 16.51
Systolic blood pressure [mmHg], mean ± SD	122.25 ± 8.97	140.00 ± 28.28
Diastolic blood pressure [mmHg], mean ± SD	78.75 ± 6.41	90.00 ± 20.00
PRA > 20 % (%)	0	25 %
HLA-mismatches, median (range)	1.5(0–4)	0.5(0–3)
Immunosuppressive treatment FK506/Pred/MMF, n (%) CsA/Pred/MMF, n (%)	75 % 25 %	75 % 25 %
Post-Tx immunologic rejection in half year,n (%)	37.5 %	0

Tx, Transplantation; HLA, Human leukocyte antigen; PRA, Panel reactive antibody; FK506, Tacrolimus; Pred, Prednisone; MMF, Mycophenolate; CsA, Cyclosporine.

The two MS repetitions identified 9268 distinct peptides corresponding to 179 unique proteins and shared an overlap of 78% of the total unique proteins of the combined data. The overlapping proteins were submitted for further analysis . Proteins from Pre-Tx, C + Tx, and Tx patients were labeled with 113, 115, and 119 tags, respectively. The ratios 113:115 and 119:115 indicate the relative abundance of proteins in Pre-Tx, Tx with respect to C + Tx. The upper and lower ranges worked out to be 2.0 and 0.5, respectively. In other words, proteins with iTRAQ ratios below the lower range were considered to be under-expressed, while those above the higher range were considered overexpressed. A total of 73 proteins showed an abundance change of at least two fold in Pre-Tx compared to C + Tx group: 39 were over-expressed and 34 were under-expressed in Pre-Tx. Meanwhile, with respect to Tx patients, 65 proteins including 36 over-expressed and 29 under-expressed proteins were detected in C + Tx.

**Figure 1:**
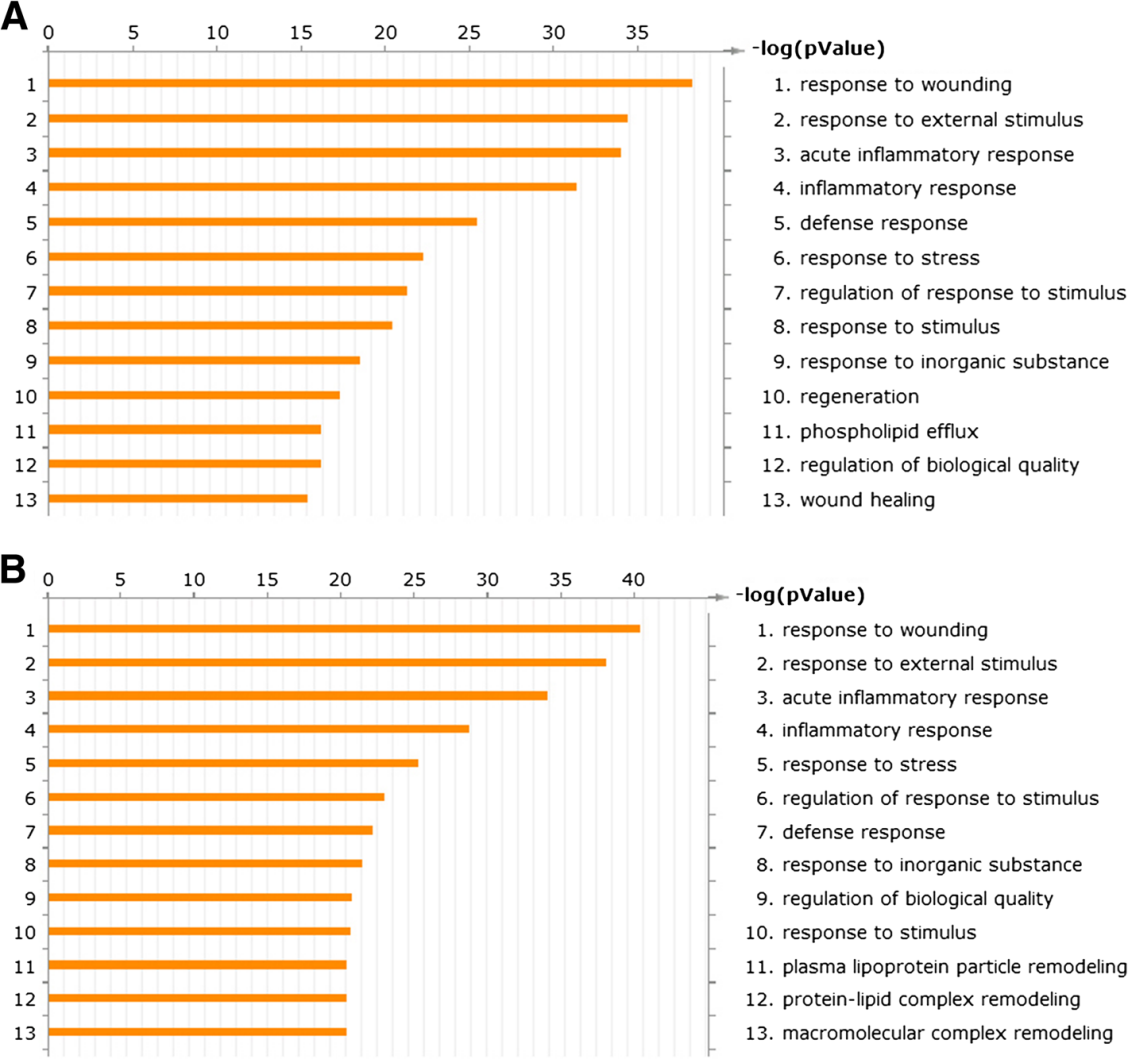
**Biologic processing of expressed proteins.** Comparison of the expressed proteins between pre-Tx and C + Tx groups (Figure 1**A**), Tx and C + Tx (Figure 1**B**) are shown. Each enumerated annotation is assigned by the enrichment score represented as *P* value. The table lists the enumerated annotations and fractions of identified proteins that belong to that particular biologic process category.

We used Metacore pathway mapping tool to analyze and build the gene ontology processes involved in the differentially expressed proteins. The differentially expressed protein between Pre-Tx and C + Tx were mapped into: 1 response to wounding; 2 response to external stimulus; 3 acute inflammatory response. Except the processes above, GO analysis showed plasma lipoprotein particle remodeling, protein-lipid complex remodeling, macromolecular complex remodeling play a role in cell therapy. Selected results are shown in Figure 1.

**Figure 2:**
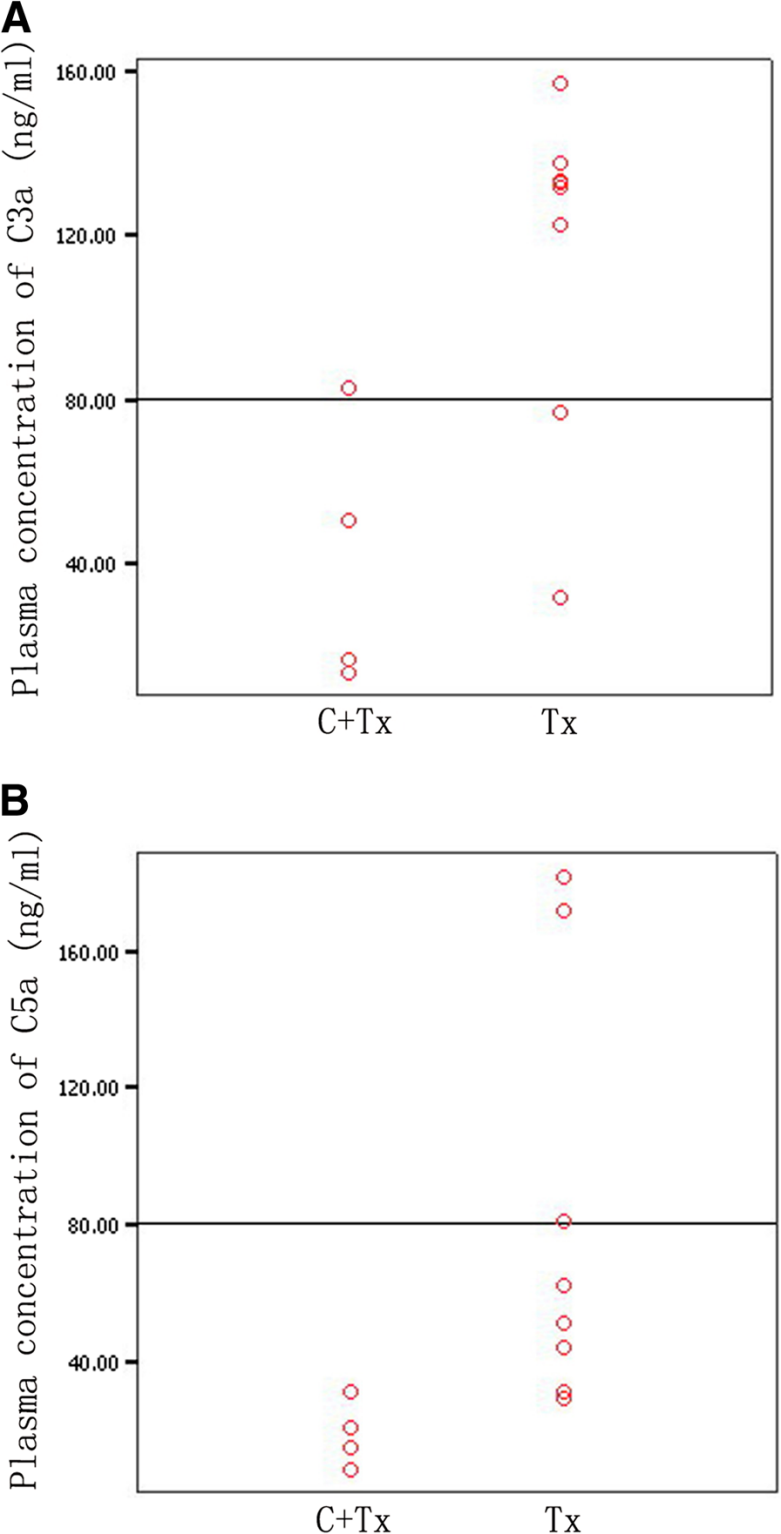
**Comparison of the plasma concentrations of complement C3a and C5a.** Plasma concentrations of complement C3a (Figure 2**A**), and C5a (Figure 2**B**) in patients of C + Tx and Tx groups. The plasma C3a and C5a concentration in Tx patients were significantly higher than those in C + Tx patients (*P* = 0.027 and 0.017, respectively).

The concentrations of complement C3a, and C5a in plasma from patients in Tx and C + Tx group are shown in Figure 2. The plasma concentrations of C3a (Figure 2A) and C5a (Figure 2B) were significantly higher in Tx patients than in C + Tx patients (C3a: Tx, median, 134.88 ng/ml; range, 31.62–157.0 ng/ml; C + Tx, median, 33.67 ng/ml; range, 13.68–83.10 ng/ml; C5a: Tx, median, 56.45 ng/ml; range, 28.82–181.46 ng/ml; C + Tx, median, 17.41 ng/ml; range, 8.36–31.07 ng/ml; *P* = 0.027 and 0.017, respectively).

**Figure 3:**
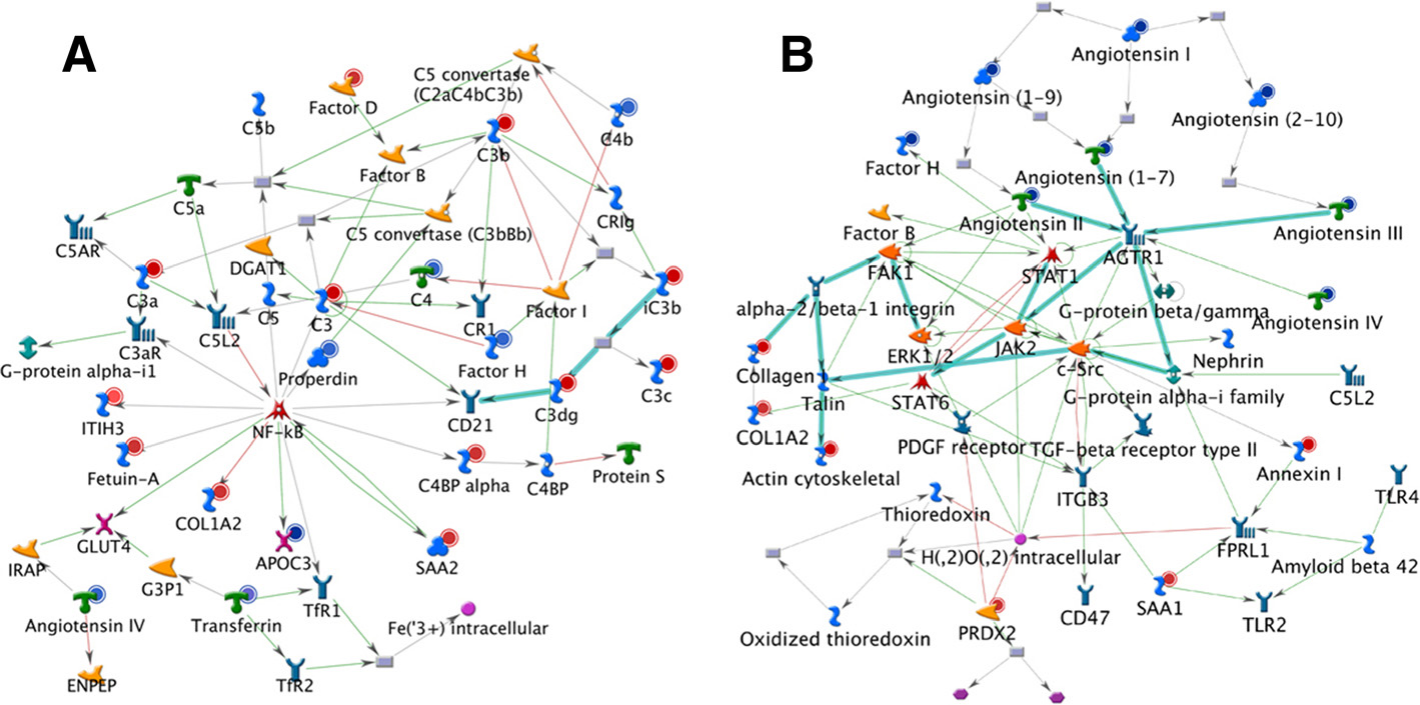
**NF-κB and STAT1 network.** Representative transcription regulatory networks based on differentially expressed data in patients with or without hematopoietic-cell transplantation using MetaCore™ network software and the Analyze Network algorithm. Two representative networks are shown: (3**A**) the NF-κB network and (3**B**) the STAT1 network. NF-κB and STAT1 are acute-phase responsive factors which are involved in the regulation of complement activation and,coagulation as well as their intercommunication. Red circles in the right corner of a gene indicate up-regulation and blue circles down-regulation.

Plasma expression level of coagulation and complement proteins was significant changed when comparing protein profile between Tx and C + Tx (Figure 3). Fibrinogen level was significantly lower in C + Tx group. The result in proteomics analysis was consistent with the findings of ELISA test in patients (Table 2). Meanwhile, a subset of lipoproteins such as protein Apolipoprotein-A1 (ApoA1), ApoC1, ApoA2, ApoE, ApoB were found to be significantly lower in Tx group compared to C + Tx group. For example, ApoA1 was 0.2 fold lower in Tx group. The expression level of ApoA1 and ApoB has been verified by using ELISA method (Table 2).

**Table 2:** Validation of differentially expressed proteins

**Variable**	**Kidney transplantation (n = 8)**	**Combined hematopoietic stem cells and kidney transplantation (n = 4)**
Apolipoprotein A1(mg/dl)	144. 13 ± 61.00	240.01 ± 62.44^*^
Apolipoprotein B(mg/dl)	78. 82 ± 23.44	133.36 ± 31.87^*^
Fibrinogen(mg/dl)	374.25 ± 28.12	278.50 ± 26.10^*^

^*^P < 0.05 vs Kidney transplantation group.

Based on differentially expressed data in patients with or without hematopoietic-cell transplantation, representative transcription regulatory networks have been developed by using MetaCore^TM^ network software and the Analyze Network algorithm. Any object containing the most connections to the root objects may represent key regulators in the clustered genes. Nuclear factor-κB (NF-κB), signal transducer and activator of transcription 3 (STAT3), signal transducer and activator of transcription 1 (STAT1) as the top three key objects found in the transcription regulatory networks. STAT1 is an acute-phase response factor and it has positive effect on complement and coagulation activation. GO processes analysis demonstrated that STAT1 is responsible for adjusting to stress (100.0%) and positive regulation of cholesterol esterification (33.3%).

## Discussion

At present, HSC transplantation is a potential method for the induction of immunologic tolerance. Although persistence of mixed chimerism may contribute to tolerance induction in combined kidney and HSC transplantation, very few clinical trials and mechanism studies are available to support this hypothesis. Through proteomic and bioinfomatic analysis of differentially expressed proteins, our study provides novel insight into the molecular events of HSC-induced immunologic tolerance.

The pre-Tx group was comprised of patients with renal failure. An imbalance of destructive and reparative cellular processes contributes to the clinicopathologic entity of renal failure. Kidney transplantation is considered the treatment of choice for many people with severe chronic kidney disease because quality of life and survival are often better than in people who use dialysis. It is evident from Figure 1A that transplantation could be considered as the biologic procedure of decreasing stress-related, immune/inflammatory, and tissue injury.

The present study reveals that lipoprotein metabolism plays a role in HSC-induced immunologic tolerance. In our study, the plasma levels of lipoproteins such as ApoA1 in C + Tx group were much higher than the patients without cell therapy compared to patients receiving HSC transplantation. A previous study demonstrated that lipoprotein abnormalities were associated with mild impairment of kidney function [11]. The protein Apo A1 was significantly lower at rejection compared with post-rejection [11]. Apo A1 may produce a plasma molecular profile which is associated with acute cellular renal allograft rejection [12]. Additionally, changes in amino acid and lipoprotein metabolism could potentially lead to alterations of the vascular wall, anorexia, endocrine dysfunction, and altered muscle intracellular signaling in chronic kidney disease. Therefore, normal lipoprotein metabolism was related with stable kidney function and decreased risk for vascular disease in chronic kidney disfunction [13].

In Figure 3A and Table 2, in comparison with Tx group, the C + Tx group exhibited a lower plasma level of fibrinogen. Given that kidney regeneration is a major predictor of outcome for patients with kidney damage, the role of fibrinogen in diagnosis, prevention, and therapeutic intervention of kidney disease has been emphasized [14]. Our previous study demonstrated that a hypercoagulable state existed in acute renal rejection patients, as evidenced by high levels of circulating fibrinogen in AR patients [8]. Fibrinogen as a soluble plasma glycoprotein may be elevated in any form of inflammation, but hyperfibrinogenemia in patients with kidney transplantation can deteriorate renal function and predict high-risk cardiovascular diseases [15]. The mechanism of rejection in solid organ transplantation is influenced by the initial inflammatory response and subsequent adaptive allo-immune response, both of which have been shown to be affected by various complement components [16]. There is growing evidence that excessive complement can be harmful to the host [17]. In Figure 3A, most complement-related proteins including C3, C3a, C3b, C3c, C3dg,C4BP were upregulated in Tx group when compared with C + Tx group; Our findings suggests HSC therapy regulates the activation of complement via both the classical (serpin peptidase inhibitor and C4BP) and the alternative pathways (complement factors B, D, H, I, and C3) but not the lectin pathway.

A crosstalk event occurs between complement and the coagulation system, apparently with the objective of enhancing local clotting and preventing microbial spread in the circulation system [18,19]. As we know, C3a and C5a play the key role in the tight regulation of activation process of the complement system [16]. Previous findings suggested [20] that an early hyper-activation of complement with generation of powerful anaphylatoxins, such as C3a and C5a, which may contribute to the disturbance of the coagulation system. Complement amplifies coagulation and inhibits fibrinolysis, mainly through C5a, which induces the expression of tissue factor [21] and plasminogen-activator inhibitor 1. In the present study, transcription regulation analysis by developing network showed NF-kB (Figure 3A), STAT1 (Figure 3B) are acute-phase responsive factors which play pivotal roles in the regulation of the complement activation and coagulation as well as their intercommunication. Which suggests in accordance with other reports [22,23] that NF-kB and STAT1 are associated with complement and coagulation cascades. Therefore, it is tempting to speculate that these significant interactions between the coagulation and complement system may play an important role after transplantation and for subsequent inflammatory and immunologic reactions. However, more validation and evidence is needed for further understanding.

This study presents a direct proteomic evidence that attenuating complement and coagulation activations is a key process in HSC-induced immunologic tolerance. The present study also indicates that stem cell transplantation contributes to the regulation of lipoprotein metabolism which is associated with kidney function. Therefore, this study provides insight into the molecular events in the HSC-induced immunologic tolerance and will help our design of new strategies for treatment.

## Competing interests

The authors declare that they have no competing interests.

## Authors’ contributions

Duojiao Wu and Guisheng Qi drafted the manuscript. Xuanchuan Wang and Ming Xu collected the clinical information and samples. Ruiming Rong helped to analyze data. Xiangdong Wang and Tongyu Zhu designed and helped to revise the manuscript. All authors read and approved the final manuscript.

## Section title

Sub-heading for section Kidney transplantation

Sub-sub heading for section Hematopoietic-cell Transplantation

Sub-sub-sub heading for section Proteomics and Bioinformatics–based analysis

## Supplementary Material

Additional file 1 **Table S1.** Clinical information of patients with cell transplantation. **Table S2.** Doses of immunosuppressive agents in patients with combined cell and kidney transplantation. **Table 3.** Transcription Factor Targets Modeling Workflow Report. Click here for file
