# Supplementary material for: Hematopoietic stem cell transplantation induces immunologic tolerance in renal transplant patients via modulation of inflammatory and repair processes
Source: J Transl Med. 2012 Aug 31;10:182. doi: 10.1186/1479-5876-10-182 (PMC3507650; doi:10.1186/1479-5876-10-182)
Supplement: Additional file 1 — Table S1. Clinical information of patients with cell transplantation. Table S2. Doses of immunosuppressive agents in patients with combined cell and kidney transplantation. Table 3. Transcription Factor Targets Modeling Workflow Report. [file 1479-5876-10-182-S1.doc]

**Supplement Table 1**

**Clinical information of patients with cell transplantation**

| **patient No.** | **Sex** | **Age** | **blood type** | **Original Disease** | **relationship** | **donor sex** | **donor age** | **donor blood type** | **CD34^+^ cells dosing(×10^6^**  **/kg)** |
| --- | --- | --- | --- | --- | --- | --- | --- | --- | --- |
| **1** | **M** | **36** | **A(+)** | **Hypertension** | **Sibling** | **M** | **41** | **A(+)** | **1.69** |
| **2** | **F** | **22** | **B(+)** | **CRF** | **M-to-C** | **F** | **46** | **O(+)** | **0.75** |
| **3** | **F** | **36** | **AB(+)** | **Nephrotic**  **syndrome** | **F-to-C** | **M** | **60** | **B(+)** | **0.31** |
| **4** | **M** | **32** | **A(+)** | **CRF** | **Sibling** | **M** | **31** | **A(+)** | **2.35** |
| **5** | **F** | **29** | **B(+)** | **CRF** | **Sibling** | **F** | **40** | **B(+)** | **0.20** |
| **6** | **M** | **33** | **A(+)** | **CRF** | **F-to-C** | **M** | **57** | **A(+)** | **3.0** |
| **7** | **M** | **31** | **A(+)** | **CRF** | **F-to-C** | **M** | **57** | **A(+)** | **2.17** |
| **8** | **M** | **37** | **B(+)** | **CRF** | **Sibling** | **F** | **40** | **B(+)** | **1.05** |
| **9** | **M** | **34** | **AB(+)** | **IgA nephropathy** | **F-to-C** | **M** | **62** | **AB(+)** | **1.05** |

**Supplement Table 2**

**Doses of immunosuppressive agents in patients with**

**combined cell and kidney transplantation**

| **patient No.** | **immunosuppressive agents** |
| --- | --- |
| **1** | **FK 506 2mg + MMF 0.5g + Pred 5mg** |
| **2** | **CsA 100mg + MMF 0.5g + Pred 2.5mg** |
| **3** | **FK 506 2mg + MMF 0.5g+Pred 2.5mg** |
| **4** | **CsA 125mg + MMF 0.5g + Pred 2.5mg** |
| **5** | **FK 506 2mg +MMF 0.5g + Pred 2.5mg** |
| **6** | **FK 506 2mg +MMF 1g + Pred 5mg** |
| **7** | **FK 506 4mg +MMF 1g + Pred 5mg** |
| **8** | **FK 506 6mg +MMF 0.75g + Pred 5mg** |
| **9** | **FK 506 3mg +MMF 1g+ Pred 5mg** |
|  |  |

**Supplement Table 3**

**Transcription Factor Targets Modeling Workflow Report**

## Selected networks built from active experiments ([TOC](#TOC_table))

The gene content of the uploaded files is used as the input list for generation of biological networks using Transcription Factor Targets Modeling algorithm with default settings. This is a variant of the shortest paths algorithm with main parameters of 1. relative enrichment with the uploaded data, and 2. relative saturation of networks with canonical pathways. These networks are built on the fly and unique for the uploaded data. In this workflow the networks are prioritized based on the number of fragments of canonical pathways on the network.

|  | **Key network objects** | **GO Processes** | **Total nodes** | **Root nodes** | **Pathways** | **p‑Value** | **zScore** | **gScore** |
| --- | --- | --- | --- | --- | --- | --- | --- | --- |
| 1 | COUP‑TFII, alpha‑2/beta‑1 integrin, DPP4, alpha‑V/beta‑5 integrin, Glycoprotein VI, alpha‑11/beta‑1 integrin, alpha‑V/beta‑3 integrin | organ development (72.9%), system development (75.7%), anatomical structure development (77.1%), multicellular organismal development (80.0%), anatomical structure morphogenesis (58.6%) | 74 | 24 | 112 | 1.87e‑52 | 76.66 | 216.66 |
| 2 | PROX1, alpha‑2/beta‑1 integrin, DPP4, alpha‑V/beta‑5 integrin, alpha‑11/beta‑1 integrin, alpha‑V/beta‑3 integrin | organ development (73.5%), anatomical structure morphogenesis (61.2%), anatomical structure development (75.5%), system development (73.5%), multicellular organismal development (77.6%) | 49 | 15 | 93 | 3.27e‑32 | 58.04 | 174.29 |
| 3 | ZNF384, alpha‑2/beta‑1 integrin, DPP4, alpha‑V/beta‑5 integrin, A2M receptor, alpha‑11/beta‑1 integrin, alpha‑V/beta‑3 integrin | organ development (68.8%), multicellular organismal development (78.1%), developmental process (78.1%), negative regulation of cellular process (62.5%), anatomical structure development (71.9%) | 68 | 23 | 30 | 8.71e‑51 | 76.74 | 114.24 |
| 4 | RXRA, alpha‑2/beta‑1 integrin, DPP4, alpha‑V/beta‑5 integrin, AGTR1, A2M receptor, alpha‑11/beta‑1 integrin, alpha‑V/beta‑3 integrin | response to external stimulus (62.8%), response to wounding (48.8%), response to stress (67.4%), negative regulation of biological process (67.4%), negative regulation of cellular process (65.1%) | 47 | 22 | 18 | 3.91e‑52 | 87.05 | 109.55 |
| 5 | KLF4, AGTR1, A2M receptor | response to external stimulus (75.9%), response to wounding (55.2%), acute inflammatory response (34.5%), inflammatory response (41.4%), regulation of inflammatory response (31.0%) | 33 | 20 | 6 | 1.56e‑50 | 94.50 | 102.00 |
| 6 | STAT3, AGTR1, A2M receptor | response to wounding (76.5%), response to external stimulus (82.4%), acute inflammatory response (47.1%), regulation of inflammatory response (47.1%), regulation of defense response (47.1%) | 21 | 15 | 2 | 1.16e‑39 | 88.86 | 91.36 |
| 7 | POLR2A, alpha‑2/beta‑1 integrin, DPP4, alpha‑V/beta‑5 integrin, alpha‑11/beta‑1 integrin, alpha‑V/beta‑3 integrin | anatomical structure development (77.4%), response to stress (67.7%), regulation of biological quality (64.5%), organ development (67.7%), developmental process (80.6%) | 31 | 14 | 16 | 5.07e‑33 | 68.19 | 88.19 |
| 8 | PAX2, A2M receptor | acute inflammatory response (47.1%), inflammatory response (58.8%), response to external stimulus (76.5%), response to wounding (64.7%), defense response (58.8%) | 17 | 13 | 0 | 4.11e‑35 | 85.61 | 85.61 |
| 9 | NF‑kB, A2M receptor | acute inflammatory response (53.3%), inflammatory response (66.7%), response to wounding (73.3%), response to external stimulus (80.0%), defense response (66.7%) | 15 | 12 | 0 | 6.90e‑33 | 84.13 | 84.13 |
| 10 | RAR‑beta/RXR‑alpha, alpha‑2/beta‑1 integrin, DPP4, alpha‑V/beta‑5 integrin, alpha‑11/beta‑1 integrin, alpha‑V/beta‑3 integrin | anatomical structure development (80.6%), response to endogenous stimulus (51.6%), response to stress (71.0%), organ development (71.0%), response to hormone stimulus (48.4%) | 31 | 13 | 16 | 3.51e‑30 | 63.31 | 83.31 |
